# Trend Analysis of Self-Harm Behaviors and Stress Management Skills in Adolescents Between 2018, 2022, and 2024: A Comprehensive Ecological Model

**DOI:** 10.3390/children12091230

**Published:** 2025-09-15

**Authors:** Tania Gaspar, Cheila Serafim, Margarida Gaspar de Matos, Marina Carvalho

**Affiliations:** 1 Digital Human-Environment Interaction Labs (HEI-LAB), Universidade Lusófona, 1749-024 Lisbon, Portugal; c.serafim019@gmail.com; 2 Comprehensive Health Research Centre (CHRC), Universidade NOVA de Lisboa, 1169-056 Lisboa, Portugal; mmmatos@ucp.pt; 3 Institute of Environmental Health (ISAMB), Faculty of Medicine, Universidade de Lisboa, 1649-028 Lisboa, Portugal; marina.carvalho@ismat.pt; 4 Laboratório Português de Ambientes de Trabalho Saudáveis, Portuguese Laboratory for Healthy Workplaces, 1400-185 Lisboa, Portugal; 5 Faculdade de Ciências Humanas (FCH), Universidade Católica Portuguesa (UCPF), 1649-023 Lisboa, Portugal; 6 ISMAT—Instituto Superior Manuel Teixeira Gomes, 8500-656 Portimão, Portugal

**Keywords:** self-harm behaviors, adolescents, trends, stress management, mental health

## Abstract

**Highlights:**

- Non-suicidal self-harm behavior (NSSHB) reflects both individual distress and systemic gaps in psychosocial support, underscoring the importance of ecological and multilevel prevention strategies.
- Stress management skills are a central vulnerability factor, consistently lower among adolescents with NSSHB, and strongly influenced by family, school, and mental well-being contexts.
- Strengthening coping resources through supportive family and school environments emerges as a crucial pathway for reducing NSSHB and promoting adolescent resilience during and beyond public health crises.

**Abstract:**

Background/Objectives: Although non-suicidal self-harming behaviors (NSSHBs) are increasingly recognized as both a symptom and risk factor in adolescent development, few studies have explored their biopsychosocial correlates, such as stress management, quality of life, family and peer support, and school-related factors, within a longitudinal framework. The present study aims to explore self-harm behaviors among adolescents from an ecological and biopsychosocial perspective over three distinct time points: pre-pandemic (2018), during the pandemic (2022), and post-pandemic (2024). Methods: The total sample comprised 12,233 adolescents, with 5695 in 2018, 5931 in 2022, and 607 in 2024. The percentage of adolescents reporting self-harm behaviors increased from 18.0% in 2018 to 21.8% in 2022 and slightly decreased to 20.2% in 2024. Results: Results show that, in both groups, stress management skills were positively predicted by family support, teacher relationship, quality of friendship, and future expectations, and they were negatively predicted by psychosomatic symptoms. The explained variance (adjusted R^2^) was consistently higher in the self-harm behavior group. The findings confirm that NSSHB is not only a symptom of individual distress but also a marker of insufficient psychosocial support and coping resources. Conclusions: Stress management skills emerged as a key vulnerability domain and were consistently lower among adolescents with NSSHB. Family support, school relationships, and mental well-being were central predictors of coping skills, reinforcing the relevance of multilevel, ecological approaches to prevention and intervention.

## 1. Introduction

Adolescents both influence and are influenced by their social and ecological contexts. Development in adolescence is considered a reciprocal process resulting from interactions between multiple systems operating at the micro-, meso-, exo-, macro-, and chronosystem levels. The quality and nature of these intersystem exchanges significantly shape developmental trajectories. Bronfenbrenner’s ecological model [1,2] highlights the importance of both endogenous adolescent processes and relational dynamics involving family, peers, school, community, and broader sociocultural forces. At the microsystem level, adolescents interact directly with key settings such as family, peers, and school. The mesosystem involves the interconnections between these immediate environments (e.g., the relationship between family and school). Within a biopsychosocial framework, both individual and contextual factors may support or hinder adolescents’ well-being and adjustment [3,4].

Non-suicidal self-harm behavior (NSSHB) is one such behavioral indicator of maladjustment and has been recognized as a significant adolescent risk behavior. NSSHB typically emerges during adolescence and tends to decline in frequency and prevalence by early adulthood. However, between 40% and 80% of youth who engage in NSSHB during adolescence may desist in early adulthood. The early onset of self-harm behaviors (before age 12) has been associated with more severe behaviors and a dual peak of incident around ages 14–15 and again between 18 and 20 [5,6,7]. NSSHB is linked to multiple psychosocial vulnerabilities, including depression, anxiety, physical symptoms, low self-concept, and strained family and peer relationships [8,9]. These behaviors are also more prevalent in school-aged youth, and schools often report limited capacity to support students who self-harm. School staff frequently feel unequipped to handle such cases [10,11], and negative attitudes toward school (e.g., low engagement and poor belonging) have been associated with elevated risk of self-harm behaviors [12].

The COVID-19 pandemic added further strain, as adolescents experienced isolation, loss of peer contact, and disruption of daily routines. These changes significantly impacted adolescents’ mental health and life satisfaction [3,13]. Research indicates that some adolescents used NSSHB as a coping mechanism to manage pandemic-related distress [14]. Recognizing the relevance of NSSHB as both a symptom and risk factor in adolescent development, it is critical to explore its biopsychosocial correlates, such as stress management skills, quality of life, family support, quality of friendship, and school-related variables, and how these may evolve over time [15].

Given the increasing recognition of non-suicidal self-harming behavior (NSSHB) as both a symptom and a significant risk factor during adolescent development, there is a pressing need to investigate its biopsychosocial correlates—such as stress management skills, quality of life, family support, peer relationships, and school-related variables—within a developmental framework. Despite growing attention to NSSHB, few studies have comprehensively examined these factors longitudinally, leaving a critical gap in our understanding of how these dimensions interact and evolve over time. This study aims to address this gap by providing an integrative, multi-dimensional analysis of NSSHB in adolescence, thereby offering novel insights into its developmental pathways and informing more targeted intervention strategies. Specifically, we analyzed the patterns of NSSHB (whether the student has ever engaged in self-harming behavior, and if so, how many times and what kind of self-harming behavior they engaged in—cutting, burning, etc.), associated risk and protective factors, and their developmental trajectories over the following three distinct time points: pre-pandemic (2018), during the pandemic (2021/22), and post-pandemic (2024). The HBSC/WHO study is conducted in 51 countries every four academic years. It was conducted in 2017/18 and 2021/22, and it will be conducted in 2025/2026. To assess whether the worsening of mental health indicators from 2017/18 to 2021/22 was largely due to the COVID-19 pandemic or whether it is more permanent, our team decided to conduct an interim study in 2023/24.

## 2. Materials and Methods

### 2.1. Participants

The total sample comprised 12,233 adolescents, with 5695 in 2018, 5931 in 2022, and 607 in 2024. Across all years, girls represented a slightly higher proportion of the sample. A general increase in reported self-harm behaviors was observed from 2018 to 2024.

Table 1 presents the descriptive characteristics of the sample across the three waves of the study (2018, 2022, and 2024), including gender, school grade, and family financial status.

### 2.2. Instrument and Procedures

This study draws on data from the 3 waves (2018, 2022 and 2024) of the Health Behavior in School-aged Children (HBSC) study [4,16,17], an international survey conducted every four years in collaboration with the World Health Organization (WHO) and governed by a standardized international protocol [18]. The survey aims to explore adolescents’ behaviors and health-related habits within their social and environmental contexts and how these factors relate to their overall health and well-being. The 2022 HBSC survey in Portugal received ethical approval from the Ethics Committee of the Lisbon Academic Medical Centre (Centro Académico de Medicina de Lisboa), the Lisbon North Hospital Centre (Centro Hospitalar Lisboa Norte), and the Directorate-General for Education and Science Statistics. Following the HBSC protocol, data collection employed a cluster sampling method, with class serving as the sampling unit. School clusters volunteered to take part in the study, and informed consent was obtained from the parents or legal guardians of all participants. Data were collected anonymously via an online platform. The variables used in this study are detailed in Table 2. Further details regarding the data collection procedures for the 2022 Portuguese edition of the HBSC study are available in Gaspar et al. [4,16,17].

Statistical analysis was performed using SPSS software version 30.0. Descriptive statistical analyses were performed to characterize the sample, Student’s t-test and ANOVA were used to compare groups, and finally, multiple linear regression analyses were performed to understand which variables best explain stress management skills for participants with and without SHB.

## 3. Results

### 3.1. Group Comparisons

Chi-square tests were conducted to examine the associations between self-harm behaviors (yes/no) and sociodemographic characteristics. Table 3 displays the percentage of adolescents with and without self-harm behaviors in each category, along with the results of the Chi-square tests.

### 3.2. Psychosocial Differences

To explore differences in psychosocial variables between adolescents with and without self-harm behaviors (SHBs), one-way ANOVA tests were conducted for each year. Table 4 presents the mean scores and standard deviations, along with F-statistics and *p*-values.

### 3.3. Multiple Linear Regression Models

Separate multiple linear regression models were conducted for adolescents with and without self-harm behaviors in each survey year (2018, 2022, and 2024). The dependent variable was stress management skills. Predictors included gender, age, family financial status, chronic illness, family support, quality of friendship, teacher relationship, future expectations, psychosomatic symptoms, and quality of life, which were previously observed to be significantly related to stress management.

To identify the predictors of stress management skills among adolescents with and without self-harm behaviors, separate multiple linear regression models were conducted for each group and year. Each model was computed in two steps as follows: the first including sociodemographic variables (gender, age, family financial status, and chronic illness), and the second incorporating psychosocial predictors (family support, quality of friendship, teacher relationship, future expectations, psychosomatic symptoms, and quality of life).

The results showed that, in both groups, stress management skills were positively predicted by family support, quality of friendship, teacher relationship, and future expectations, and they were negatively predicted by psychosomatic symptoms. The explained variance (adjusted R^2^) was consistently higher in the self-harm behavior group.

In the case of the models for adolescents without self-harm behaviors, the 2018 model showed an explained variance of 34% (F = 175.002 (10, 3438), *p* < 0.001), the 2022 model showed an explained variance of 46% (F = 378.194 (10, 4371), *p* < 0.001), and the 2024 model showed an explained variance of 52% (F = 21.221 (10, 194), *p* < 0.001). With regard to the models of adolescents with self-harm behaviors, we found that the 2018 model showed an explained variance of 37% (F = 44.357 (10, 745), *p* < 0.001), the 2022 model showed an explained variance of 40% (F = 74.879 (10, 1144), *p* < 0.001), and the 2024 model showed an explained variance of 79% (F = 11.053 (10, 30), *p* < 0.001).

Across all waves (Table 5), the models were significant for both groups, explaining a larger proportion of variance among adolescents with self-harm behaviors (adjusted R^2^ ranging from 0.370 to 0.521) compared to those without (adjusted R^2^ ranging from 0.335 to 0.498). Psychosocial factors, particularly family support and psychosomatic symptoms, consistently emerged as strong predictors of adolescents’ stress management skills. Across all years, the models for adolescents with self-harm behaviors consistently explained more variance in stress management skills than for those without self-harm behaviors. The strongest predictors for both groups included psychosomatic symptoms (negative predictor), family support, and teacher relationship (positive predictors). For adolescents with self-harm behaviors, psychosocial resources such as quality of friendship and teacher relationship appear to be especially relevant.

## 4. Discussion

This study analyzed trends in non-suicidal self-harm behavior (NSSHB) among Portuguese adolescents using HBSC data collected across three waves (2018, 2022, and 2024), adopting a biopsychosocial and ecological framework. The findings highlighted relevant changes over time and offer important insights into the psychosocial correlates of NSSHB before, during, and after the COVID-19 pandemic.

Consistent with prior research [22,23], the prevalence of self-harm behaviors was higher among girls and appeared to peak during adolescence. Although an increase in NSSHB was observed during the pandemic (from 18.0% in 2018 to 21.8% in 2022), the rate slightly declined in 2024 (20.2%), suggesting some recovery post-pandemic but remaining above pre-pandemic levels. These trends are in line with evidence that adolescents experienced greater psychological vulnerability and reduced access to social support during lockdowns [3,14]. Across all years, adolescents engaging in NSSHB reported significantly poorer outcomes in stress management skills, quality of life, psychosomatic symptoms, family support, quality of friendship, and teacher relationship. These findings confirm that NSSHB is closely linked to emotional dysregulation and interpersonal difficulties [9,22]. The consistent gender effect—higher NSSHB rates among girls—may be related to internalizing symptomatology and body image concerns being more prevalent among female adolescents [24,25].

One of the most salient findings was the consistent and large gap in stress management skill competencies between adolescents with and without NSSHB. These results corroborate studies showing that poor stress coping skills are a central vulnerability factor for self-harm behavior [26,27]. Adolescents with limited coping strategies may resort to NSSHB as a maladaptive emotion regulation strategy [28].

The regression analyses further clarified that stress management skills are influenced by a combination of sociodemographic and psychosocial factors. Across all years, the strongest positive predictors were family support, teacher relationship, and future expectations, while psychosomatic symptoms emerged as the most consistent negative predictor. These results are in line with previous studies emphasizing the protective role of supportive family and school environments [17,29,30]. Importantly, the models explained a greater proportion of variance in stress management skills among adolescents with NSSHB (adjusted R^2^ up to 0.52) compared to those without, suggesting that psychosocial resources may be even more critical for adolescents experiencing psychological distress. Notably, the influence of gender was more pronounced in 2024, where girls with NSSHB showed significantly lower stress management capacities.

Our findings offer several innovative contributions to the literature on adolescent non-suicidal self-harm (NSSH) and stress management. By demonstrating that psychosocial resources, especially family support, teacher relationship, quality of friendships, and future expectations, positively predict stress management skills while psychosomatic symptoms have a negative impact, we shed light on the intricate biopsychosocial dynamics underpinning adaptive coping. Crucially, the models accounted for more variance among adolescents who engaged in self-harm (adjusted R^2^ 0.370–0.521) than those who did not (0.335–0.498), underscoring the salience of psychosocial predictors in this high-risk subgroup.

Integrating an ecological perspective, our results align with Bronfenbrenner’s framework by highlighting that adolescents’ coping capacity is influenced by intersecting social environments. The stronger explanatory power of psychosocial factors among those with self-harm behaviors points to heightened sensitivity to protective relationships and symptom loads when coping is most critical. This nuance is often missing from cross-sectional studies and non-longitudinal designs.

Guided by Bronfenbrenner’s ecological model [1,2], the findings highlight the relevance of multiple system levels—from intrapersonal skills like emotional regulation to microsystem-level support from family, friends, and teachers. During the pandemic, disruptions to school and friendship contexts may have weakened protective buffers, amplifying NSSHB risk [16,17,31]. In contrast, the post-pandemic improvement in some indicators (e.g., friendships) may reflect the partial reactivation of these support systems [13,32].

## 5. Conclusions

This study provides an updated and comprehensive picture of non-suicidal self-harm behavior (NSSHB) trends among Portuguese adolescents before, during, and after the COVID-19 pandemic, using HBSC data from 2018, 2022, and 2024. By analyzing psychosocial predictors and differentiating adolescents with and without NSSHB, this research contributes significantly to understanding the multiple determinants of stress management skills—a core competence strongly associated with self-harm behaviors.

The findings confirm that NSSHB is not only a symptom of individual distress but also a marker of insufficient psychosocial support and coping resources. Stress management skills emerged as a key vulnerability domain and were consistently lower among adolescents with NSSHB. Family support, school relationships, and mental well-being were central predictors of coping skills, reinforcing the relevance of multilevel, ecological approaches to prevention and intervention.

### Limitations, Future Directions, and Implications for Practice

Although the study has a random and representative sample, with cross-sectional studies at three different points in time, it relies on self-reports, which may be subject to recall and social desirability bias. The cross-sectional nature of the data also limits causal interpretations. Future longitudinal studies should investigate mediating and moderating pathways (e.g., emotional regulation and social support quality) and explore the long-term outcomes of adolescents with NSSHB.

This cross-sectional, quantitative study is part of a larger study with a random sample representative of Portuguese students. One limitation of this approach is that the instrument used is more general in nature, focusing on students’ health in general rather than specifically on self-harm. The study involves three relevant moments in the context of the pandemic, but as it is a cross-sectional study, it does not follow the same students across these three moments. However, the robustness of the methodology and the relevance of having a random sample are highly valuable and illustrate that the impact of the pandemic was very strong and that this impact remains two years after the pandemic began. This result points to the fundamental importance of promoting mental health and preventing self-harming behaviors in children and adolescents.

Given the strong association between NSSHB and psychosocial vulnerabilities, school-based interventions should prioritize strengthening adolescents’ coping skills, emotional literacy, and access to trusted and supportive adults. Enhancing family–school collaboration may also contribute to early identification and more effective responses to distress in youth.

We propose that future studies conduct a longitudinal study of self-harming behavior and its severity (students who do not self-harm, students who try it once, and students who engage in self-harming behavior more than once). Qualitative studies with in-depth interviews and focus groups would help to better understand students’ beliefs and motivations for resorting to self-harming behavior. The present HBSC/WHO study is conducted in 51 countries. It would be important to conduct an in-depth study of self-harming behavior from an ecological perspective and to verify similarities and differences related to cultural and regional issues.

The recommendations for practice, given the developmental sensitivity of adolescence, are early and sustained action across sectors to prevent the escalation of self-harm behaviors and promote recovery.

The recommendations should take into account the ecological perspective, including individual recommendations for children and adolescents, for the family and school context and the respective actors, and for the community and political authorities.

For children and adolescents, the study highlights the need to promote emotional literacy, problem-solving skills, and positive self-regulation strategies from an early age. They need to be attentive to early warning signs of distress, such as psychosomatic symptoms, withdrawal, or irritability; seek support from professionals when needed; and participate actively in prevention or treatment processes [33].

For the school and community context, they need to facilitate access to safe, confidential support spaces. They should reduce stigma around mental health and help-seeking behaviors through peer-led and youth-participatory programs. In relation to parents and families, we emphasize the need for strengthening open communication and emotional availability in the family environment [34].

Focusing on teachers and schools, the study reinforces the priority of providing training on how to recognize and respond to emotional distress and self-harm behaviors in students. They need to foster a supportive and inclusive school climate that promotes psychological safety and peer connection; integrate social–emotional learning into the curriculum; and offer structured support interventions when needed [35,36].

Finaly for policy makers, we stress the fundamental importance of ensuring sustainable investment in school-based mental health services and youth psychosocial support networks. They need to develop national strategies for the prevention of self-harm behaviors, grounded in evidence-based and rights-based approaches, and promote multisectoral collaboration (education, health, youth, and social protection) for coordinated intervention models [37].

By recognizing self-harm behaviors as a multifactorial phenomenon, this study advocates for systemic, integrated responses that empower adolescents and enhance their environments to support their healthy emotional development. Future research and policy should focus not only on reducing risk but also on strengthening protective systems around youth.

## Figures and Tables

**Table 1:** Sample characteristics by year of data collection (2018, 2022, and 2024) (*N* = 16,521).

Variable	Category	2018 (%)	2022 (%)	2024 (%)
Gender	Boys	46.1	45.3	49.1
	Girls	53.9	54.7	50.9
School grade	8th Grade	48.6	32.8	23.6
	10th Grade	30.0	36.2	33.8
	12th Grade	21.4	31.0	47.7
Family financial status (2 levels)	Low	54.4	48.3	57.2
	High	45.6	51.7	42.8
Self-harm behaviors	Yes	18.0	21.8	20.2
	No	82.0	78.2	79.8

Note: Values represent valid percentages after exclusion of system-missing data.

**Table 2:** Description of variables and measures according to the international HBSC study protocol [17].

Variable	Measure	Interpretation	α (a)	α (b)	References
Gender	Single item.	Dichotomic variable 1—boy; 2 girl.	–	–	[17]
Age	Single item.	Continuous variable.	10	18	[17]
School grade	Single item.	2 = 8th grade; 3 = 10th grade; 4 = 12th grade; etc.	–	–	[17]
Stress management skills	4-item scale (5-point Likert). Example: “How often have you felt unable to control important things?”	Higher scores = better stress management. Range: 4–20.	0.85	0.70	[17,19]
Physical symptoms	5-item scale (5-point Likert). Example: “Headaches.”	Higher scores = fewer symptoms. Range: 5–25.	0.72	0.77	[3,4]
Psychological symptoms	4-item scale (5-point Likert). Example: “Nervousness.”	Higher scores = fewer symptoms. Range: 4–20.	0.78	0.84	[3,4]
Quality of life	10-item scale (5-point Likert). Example: “Have you been feeling well?”	Higher scores = greater well-being. Range: 10–50.	0.86	0.84	[17,20]
Chronic illness	Single item.	1 = No; 2 = Yes.	–	–	[17]
Teacher relationship	3-item scale (5-point Likert). Example: “I feel my teachers accept me.”	Higher scores = poorer relationship. Range: 3–15.	0.83	0.83	[17]
Self-harm behaviors	Single item.	1 = No self-harm; 2 = 1 time; 3 = 2 + times.	–	–	[17]
Family support	4-item scale (7-point Likert). Example: “My family really tries to help.”	Higher scores = greater support. Range: 4–28.	0.94	0.94	[17]
Family financial status	Single item.	1 = Very well; 2 = Well; 3 = Average; 4 = Poorly; 5 = Very poorly; 6 = Don’t know. Dichotomized: High (1–3) vs. Low (4–5).	–	–	[17]
Future expectations	10-step Cantril ladder (1–10).	Higher scores = more positive expectations.	–	–	[17,21]
Quality of friendship	10-step Cantril ladder (1–10).	Higher scores = better friendship quality.	–	–	[17,21]

**Table 3:** Group comparisons of self-harm behaviors (SHBs) by sociodemographic variables using Pearson’s Chi-square test.

Variable	Category	2018 (%)	2022 (%)	2024 (%)	*χ* ^2^	*p*
Gender	Boys (SHB)	43.4	33.8	34.4	2.30	0.130
	Girls (SHB)	56.6	66.2	65.6	77.64	<0.001
School grade	8th (SHB)	56.9	40.7	37.6	50.70	<0.001
	10th (SHB)	27.0	37.1	28.6	76.72	<0.001
	12th (SHB)	16.2	22.1	33.8	5.68	0.128
Family financial status (2 levels)	Low (SHB)	56.6	55.6	61.7	1.83	0.176
	High (SHB)	43.4	44.4	38.3	33.84	<0.001
Chronic Illness	Yes (SHB)	19.7	24.1	48.1	9.23	0.002
	No (SHB)	80.3	75.9	51.9	23.28	<0.001

Note: SHB = Self-harm behavior. Percentages refer to the distribution within each year and category of self-harm behaviors. Full statistics available upon request.

**Table 4:** Mean differences in psychosocial indicators by self-harm behavior (SHB) status (ANOVA).

Variable	Year	No SHB (*M* ± *SD*)	SHB (*M* ± *SD*)	*F*	*p*
Future Expectations	2018	7.33 ± 1.84	6.75 ± 2.21	63.03	<0.001
	2022	7.28 ± 1.92	6.21 ± 2.41	278.42	<0.001
	2024	7.51 ± 2.04	6.26 ± 2.43	36.60	<0.001
Quality of Friendship	2018	8.63 ± 1.65	7.92 ± 2.23	105.45	<0.001
	2022	8.36 ± 1.85	7.60 ± 2.22	157.37	<0.001
	2024	8.53 ± 1.58	7.10 ± 2.28	71.45	<0.001
Stress Management Skills	2018	3.33 ± 0.64	2.87 ± 0.66	327.29	<0.001
	2022	3.33 ± 0.71	2.69 ± 0.74	803.12	<0.001
	2024	3.30 ± 0.74	2.60 ± 0.72	58.60	<0.001
Psychosomatic Symptoms	2018	2.01 ± 0.77	2.55 ± 0.95	329.15	<0.001
	2022	2.17 ± 0.85	3.03 ± 0.96	962.17	<0.001
	2024	2.22 ± 0.86	3.16 ± 0.89	125.66	<0.001
Quality of Life	2018	3.73 ± 0.69	3.24 ± 0.79	347.70	<0.001
	2022	3.71 ± 0.65	3.09 ± 0.68	880.25	<0.001
	2024	3.69 ± 0.68	2.99 ± 0.62	117.56	<0.001
Teacher Relationship	2018	3.67 ± 0.80	3.44 ± 0.93	57.03	<0.001
	2022	3.74 ± 0.79	3.37 ± 0.89	205.50	<0.001
	2024	3.62 ± 0.85	3.37 ± 0.86	9.38	0.002
Family Support	2018	5.97 ± 1.52	5.01 ± 1.98	259.51	<0.001
	2022	5.82 ± 1.51	4.47 ± 1.90	705.99	<0.001
	2024	5.47 ± 1.57	4.41 ± 1.50	49.53	<0.001

Note: SHB = Self-harm behavior. All F-tests were significant at *p* < 0.01 level unless otherwise stated.

**Table 5:** Linear regression model predicting stress management skills by self-harm behavior group and survey year.

Predictor	No SHB (B)	SE	*β*	*t*	SHB (B)	SE	*β*	*t*
2018								
Age	−0.05	0.02	−0.05 **	−2.92	−0.06	0.04	−0.06	−1.52
Gender (girl)	−0.24	0.03	−0.12 ***	−7.41	−0.28	0.06	−0.13 ***	−4.82
Family financial status (high)	0.06	0.02	0.04 **	3.14	0.04	0.04	0.03	1.02
Chronic illness	−0.10	0.03	−0.04 ***	−3.32	−0.12	0.06	−0.05	−1.91
Family support	0.12	0.01	0.20 ***	12.20	0.14	0.02	0.25 ***	6.85
Quality of friendship	0.08	0.01	0.11 ***	8.13	0.10	0.02	0.12 ***	4.23
Teacher relationship	0.10	0.01	0.12 ***	8.40	0.12	0.02	0.13 ***	4.80
Future expectations	0.06	0.01	0.08 ***	6.02	0.08	0.02	0.10 **	3.15
Psychosomatic symptoms	−0.19	0.01	−0.26 ***	−15.3	−0.22	0.02	−0.30 ***	−9.45
Quality of life	0.15	0.02	0.13 ***	8.52	0.18	0.03	0.16 ***	5.69
2022								
Age	−0.07	0.01	−0.07 ***	−5.71	−0.04	0.02	−0.04	−1.81
Gender (girl)	−0.36	0.02	−0.19 ***	−15.23	−0.31	0.03	−0.14 ***	−9.97
Family financial status (high)	0.05	0.01	0.04 ***	4.45	0.03	0.02	0.03	1.30
Chronic illness	−0.18	0.02	−0.09 ***	−9.11	−0.21	0.03	−0.09 ***	−6.81
Family support	0.14	0.01	0.23 ***	19.00	0.17	0.01	0.30 ***	13.50
Quality of friendship	0.07	0.01	0.10 ***	8.62	0.08	0.01	0.10 ***	6.44
Teacher relationship	0.12	0.01	0.14 ***	12.26	0.15	0.01	0.17 ***	9.80
Future expectations	0.07	0.01	0.09 ***	8.51	0.09	0.01	0.11 ***	6.53
Psychosomatic symptoms	−0.22	0.01	−0.30 ***	−21.9	−0.25	0.01	−0.35 ***	−15.6
Quality of life	0.12	0.01	0.11 ***	10.45	0.15	0.01	0.14 ***	9.97
2024								
Age	−0.03	0.02	−0.03	−1.36	−0.01	0.05	−0.01	−0.19
Gender (girl)	−0.41	0.07	−0.25 ***	−6.12	−0.33	0.11	−0.20 **	−3.05
Family financial status (high)	0.09	0.03	0.09 **	2.88	0.06	0.05	0.07	1.23
Chronic illness	−0.26	0.07	−0.12 **	−3.71	−0.19	0.11	−0.08	−1.67
Family support	0.16	0.03	0.31 ***	5.32	0.18	0.04	0.35 ***	4.42
Quality of friendship	0.03	0.03	0.06	1.12	0.05	0.04	0.10	1.34
Teacher relationship	0.10	0.03	0.17 **	3.09	0.12	0.05	0.21 *	2.43
Future expectations	0.04	0.01	0.07 *	2.07	0.06	0.02	0.09	1.80
Psychosomatic symptoms	−0.23	0.03	−0.32 ***	−6.98	−0.28	0.05	−0.36 ***	−5.27
Quality of life	0.09	0.03	0.09 *	2.54	0.10	0.05	0.11 *	2.12

Note: SHB = Self-harm behavior. * < 0.05; ** < 0.01; *** < 0.001.

## Data Availability

Data are unavailable due to privacy or ethical restrictions.
